# The microRNA let-7b-5p Is Negatively Associated with Inflammation and Disease Severity in Multiple Sclerosis

**DOI:** 10.3390/cells10020330

**Published:** 2021-02-05

**Authors:** Georgia Mandolesi, Francesca Romana Rizzo, Sara Balletta, Mario Stampanoni Bassi, Luana Gilio, Livia Guadalupi, Monica Nencini, Alessandro Moscatelli, Colleen Patricia Ryan, Valerio Licursi, Ettore Dolcetti, Alessandra Musella, Antonietta Gentile, Diego Fresegna, Silvia Bullitta, Silvia Caioli, Valentina Vanni, Krizia Sanna, Antonio Bruno, Fabio Buttari, Chiara Castelli, Carlo Presutti, Francesca De Santa, Annamaria Finardi, Roberto Furlan, Diego Centonze, Francesca De Vito

**Affiliations:** 1 Synaptic Immunopathology Lab, IRCCS San Raffaele Pisana, 00166 Rome, Italy; georgia.mandolesi@uniroma5.it (G.M.); livia.guadalupi@gmail.com (L.G.); monicanencini@hotmail.com (M.N.); alessandra.musella@uniroma5.it (A.M.); antonellag79@gmail.com (A.G.); diego.fresegna@gmail.com (D.F.); silvia.bullitta88@gmail.com (S.B.); valentina_vanni@hotmail.it (V.V.); 2 Department of Human Sciences and Quality of Life Promotion, University of Rome San Raffaele, 00166 Rome, Italy; 3 Department of Systems Medicine, Tor Vergata University, 00133 Rome, Italy; f.rizzo@med.uniroma2.it (F.R.R.); balletta.sara@gmail.com (S.B.); a.moscatelli@hsantalucia.it (A.M.); colleen.patriciaR@gmail.com (C.P.R.); ettoredolcetti@hotmail.it (E.D.); krizia.sanna@live.it (K.S.); brunoa.neuro@gmail.com (A.B.); 4 Unit of Neurology, IRCCS Neuromed, 86077 Pozzilli, Italy; m.stampanonibassi@gmail.com (M.S.B.); luana.gilio@gmail.com (L.G.); silviacaioli@yahoo.it (S.C.); fabio.buttari@gmail.com (F.B.); f.devito.molbio@gmail.com (F.D.V.); 5 Laboratory of Neuromotor Physiology, IRCCS Fondazione Santa Lucia, 00143 Rome, Italy; 6 Laboratory of Functional Genomics and Proteomics of Model Systems, Department of Biology and Biotechnologies “C. Darwin,” University of Rome “Sapienza”, Rome 00185, Italy; valerio.licursi@uniroma1.it (V.L.); calyme95@gmail.com (C.C.); carlo.presutti@uniroma1.it (C.P.); 7 Institute of Biochemistry and Cell Biology (IBBC), National Research Council of Italy (CNR), 00015 Rome, Italy; francesca.desanta@cnr.it; 8 Neuroimmunology Unit, Institute of Experimental Neurology (INSpe), Division of Neuroscience, San Raffaele Scientific Institute, 20132 Milan, Italy; finardi.annamaria@hsr.it (A.F.); furlan.roberto@hsr.it (R.F.)

**Keywords:** multiple sclerosis (MS), miRNAs, let-7, inflammation, Expanded Disability Status Scale (EDSS), progressive multiple sclerosis (PMS), RANTES, IL5, G_CSF

## Abstract

The identification of microRNAs in biological fluids for diagnosis and prognosis is receiving great attention in the field of multiple sclerosis (MS) research but it is still in its infancy. In the present study, we observed in a large sample of MS patients that let-7b-5p levels in the cerebrospinal fluid (CSF) were highly correlated with a number of microRNAs implicated in MS, as well as with a variety of inflammation-related protein factors, showing specific expression patterns coherent with let-7b-5p-mediated regulation. Additionally, we found that the CSF let-7b-5p levels were significantly reduced in patients with the progressive MS compared to patients with relapsing-remitting MS and were negatively correlated with characteristic hallmark processes of the two phases of the disease. Indeed, in the non-progressive phase, let-7b-5p inversely associated with both central and peripheral inflammation; whereas, in progressive MS, the CSF levels of let-7b-5p negatively correlated with clinical disability at disease onset and after a follow-up period. Overall, our results uncovered, by the means of a multidisciplinary approach and multiple statistical analyses, a new possible pleiotropic action of let-7b-5p in MS, with potential utility as a biomarker of MS course.

## 1. Introduction

Multiple sclerosis (MS) is a chronic inflammatory, demyelinating and neurodegenerative disease of the central nervous system (CNS), characterized by a highly variable relapse rate and a progressive increase of clinical disability. Although its etiology remains elusive, it is well known that MS is a multifactorial disease caused by a complex gene-environment interaction. The pathological hallmark of MS is a progressive blood-brain barrier (BBB) disruption that promotes infiltration of peripheral immune cells in the CNS, leading to an autoimmune response against myelin antigens [1]. Indeed, high levels of T cells and related cytokines and chemokines have been found both in the CNS lesions and in the cerebrospinal fluid (CSF) of patients with MS, thus contributing to gliosis, inflammation, demyelination, synaptopathy, and finally neuroaxonal degeneration [2]. The inflammatory events are typical in the relapsing-remitting (RRMS) phase of the disease, during which there is a full or partial recovery of clinical symptoms until reaching a phase of irreversible progressive worsening of the disease (i.e., secondary progressive MS, SPMS). However, a small number of patients with MS enter directly into the progressive phase after clinical onset (i.e., primary progressive MS, PPMS) due to irreversible accumulation of neurological disabilities as a result of axonal injury and neuronal loss [3]. 

The identification of biomarkers, as measurable indicators of pathogenic processes and as tools to discern clinical MS phenotypes, has recently gained great attention but it is still a critical issue under investigation. Among the biological fluids, the CSF is the main source of biomarkers representing a valid means through which it is possible to predict the disease course and the individual response to treatment [4,5]. 

Recently, the small non-coding RNAs (miRNAs) are emerging as important modulators of gene expression and have been found also in the CSF [5,6]. These molecules are encoded by a conserved class of genes across animals, and their mature products are constituted by single-stranded RNAs, approximately 22 nucleotides in length, repressing post-transcriptionally the translation of target mRNAs through an imperfect base pairing [7]. MiRNAs are able to directly regulate multiple targets and a single mRNA can be targeted by many miRNAs [8], thus controlling through a pleiotropic action different cellular processes and mechanisms involved in development, homeostasis, and disease [9,10].

In the last decade, neuroinflammation has shown to be one of the major processes regulated by miRNAs. Thus, a better understanding of miRNA dysregulation has the enormous potential to develop promising and novel therapeutic targets to personalized treatment, and to rapidly expand fields in MS biomarker research [11]. 

It has been recently suggested that the let-7 family of miRNAs, known as crucial regulators of developmental processes and cancer, may modulate the inflammatory response within the CNS in various neurodegenerative diseases [12,13,14,15,16,17]. Moreover, an emerging role for the let-7 family in MS pathophysiology has just started to be dissected, despite the very few clinical studies available [13,18,19]. 

In the present article, we aimed to provide new insights of let-7 family involvement in MS. Indeed, we explored the levels of the most representative members of the family (let-7b-5p, let-7e-5p, let-7f-5p) in the CSF of a large cohort of patients with MS and we found that the let-7b-5p was highly correlated to inflammatory processes linked to disease, disease stage, and disability in MS.

## 2. Materials and Methods

### 2.1. Let-7 Target mRNA Analysis and Gene Ontology Enrichment Analysis

A list of target mRNAs of let-7 family was downloaded from the MIENTURNET webtool [20] using only experimentally validated targets with strong evidence in humans (by report assays, qPCR and western blot analysis) from miRTarBase (http://mirtarbase.cuhk.edu.cn/php/index.php) [21]. Then, functional enrichment analysis of let-7 family targets was performed using the Bioconductor R package clusterProfiler v3.14.3 [22] with annotation of Gene Ontology Database, Biological Process categories [23].

Finally, the enriched GO categories were grouped in three main processes crucial in MS pathophysiology as follow: Inflammation [1]: GO:0042110, GO:0045787, GO:0007259, GO:0048872, GO:0042035, GO:0042089, GO:0002683, GO:0001818, GO:0002544, GO:0032496, GO:0002688, GO:0071560, GO:0150076, GO:0034612, GO:1905517, GO:0061900, GO:0045071, GO:1905521, GO:2000551, GO:0150077, GO:1905523, GO:0140131, GO:0002440. Neuronal homeostasis [17,24,25]: GO:0070997, GO:0006979, GO:0007611, GO:0048169, GO:0106056, GO:0110053, GO:0099149, GO:0099177, GO:0097720, GO:0106057, GO:0098884, GO:0140239, GO:0042136, GO:0099072, GO:0099590. MiRNA biosynthesis and functioning [5,26,27]: GO:0035196, GO:0060148, GO:0043488.

### 2.2. Clinical Study Design

This observational prospective study included 166 patients with MS (Clinically Isolated Syndrome/Radiologically Isolated Syndrome, CIS/RIS, n = 25; Relapsing-Remitting multiple sclerosis, RRMS, n = 117; Progressive multiple sclerosis, PMS, n = 24) as the main cohort. Twenty age- and sex-matched control subjects were recruited as in [28]. For details on demographic characteristics see Table 1 (all patients). All patients with MS were treatment-naïve, and CSF withdrawal was performed at least 3 months after the last corticosteroid therapy. After patient admittance to the neurological department of IRCCS Neuromed Hospital (T0), all subjects underwent neurological assessment, conventional brain MRI scan and CSF withdrawal performed in sequence and within 24h, according to Italian standard clinical practice. A subgroup of patients with non-progressive MS (non-PMS: CIS/RIS and RRMS) were also subjected to cognitive evaluation.

During the follow-up, neurological assessment was repeated after 1 year (Tf1) if no relapse occurred. 

CSF levels of 24 miRNAs and 27 inflammation-related biochemical parameters (see next sections) were detected at T0 in all subjects enrolled in the main cohort of the study. To confirm hierarchical clustering analysis for inflammatory protein factors (see next sections), their quantification was extended to other 107 patients with MS, who represent the extended cohort (CIS/RIS, n = 49; RRMS, n = 180; PMS, n = 44). See the Supplementary Table S1 for details.

This study was approved by the Institutional Review Board (CE 26 October 2017; NCT03217396 recorded in https://clinicaltrials.gov/) of the IRCCS Istituto Neurologico Mediterraneo (INM) Neuromed, Pozzilli (Isernia, Italy), according to the Declaration of Helsinki principles. Written informed consent was signed by all subjects.

### 2.3. Patients with MS 

Patients with MS were included in the study with the following eligibility criteria: (i) diagnosis of multiple sclerosis according to the 2010 McDonald criteria [29]; (ii) EDSS score ≤7 at T0; age ≥18 and ≤65 years (inclusive); (iii) no immunomodulatory or immunosuppressive treatment before the CSF withdrawal; (iv) ability to provide written informed consent. Additional exclusion criteria were: (i) EDSS score >7 at T0; (ii) age <18 or >65 years; (iii) comorbidities for neurological diseases other than MS (i.e, Parkinson disease, Alzheimer disease, stroke); (iv) history or presence of any unstable medical condition, such as malignancy or infection that might confound the results of the study; (v) pregnancy or lactation; (vi) inability to provide written informed consent.

#### Clinical Parameters

For each patient, the following demographic and clinical variables were considered and analyzed: sex (F/M); age (in years); disease duration, estimated as the number of months from onset to the most recent assessment of disability; clinical disability, assessed by Expanded Disability Status Scale (EDSS); Progression Index (PI = EDSS/disease duration in months). Disease activity included clinical and/or radiological activity, evaluated by MRI scans. Conventional MRI scans (1.5 Tesla) were performed according to Italian standard clinical practice and the radiological activity was assessed according to [29]. Peripheral blood samples were collected from patients with MS by standard venipuncture EDTA collection tubes (Vacutainer^®^, Becton Dickinson, Milan, Italy) and lymphocyte counts were performed as described in Stampanoni Bassi et al., 2020 [30]. Two verbal fluency tests were performed to assess the cognitive functions [31] in a subgroup of patients with non-PMS, without any signs of dementia evaluated by Mini Mental State Examination (MMSE). Specifically, for the semantic fluency assessment (categorical memory function), patients with a MMSE score >23.8 [32] were asked to say as many words as possible belonging to the ‘‘colors’’, ‘‘animals’’ and ‘‘fruits’’ categories in three different trials, which also lasted 60 s each. To evaluate the phonemic fluency (executive function), patients were asked to generate as many words as possible beginning with the letters ‘‘A’’, ‘‘F’’ and ‘‘S’’ in three different trials, each lasting 60 s. In both tasks, the greater the number of pronounced words, the better was the patient’s cognitive performance. The results were corrected for gender, age and education according to [31]. 

### 2.4. RNA Extraction from CSF and miRNA Detection 

Briefly, after the collection of CSF samples (0.5–2 mL), cellular elements were removed immediately by centrifugation (1300 rpm, 10 min) and supernatants were stored at −80 °C, as previously described [28]. Immediately before extraction performed with miRNeasy Micro Kit (QIAGEN, Hilden, Germany), 1 μg of carrier RNA (MS2 RNA) and 2 fmol of synthetic spike-in (cel-miR-39-3p) were added, respectively, to increase RNA yield and to control efficiency of extraction, as recommended by Exiqon for subsequent applications. Mandatorily, RNA samples were reverse transcribed the same day of the extraction. Then, the levels of 24 miRNAs were detected using miRCURY LNA Universal RT microRNA PCR system and custom Pick-&-Mix microRNA PCR Panels with selected dried down microRNA LNA PCR primer sets (Exiqon, QIAGEN), according to the manufacturer’s protocol, on a 7900 Fast Real Time PCR system (Applied Biosystems, Thermo Fisher, Waltham, MA USA). As endogenous reference for the ΔCt calculation (Ct miR–Ct miR-204-5p), miR-204-5p was identified among the detected miRNAs by using the algorithm GeNorm, run in R [33,34] (Supplementary Figure S1), in accordance with previous independent studies [35,36]. Low ΔCt-values indicate high miR levels and data are presented as 2^−ΔCt^.

This is the list of the miRNAs included in the screening: let-7b-5p, let-7e-5p, let-7f-5p, miR-9-5p, miR-16-5p, miR-21-5p, miR-24-3p, miR-34a-5p, miR-34c-5p, miR-92a-3p, miR-124-3p, miR-132-3p, miR-135a-5p, miR-142-3p, miR-146a-5p, miR-146b-5p, miR-150-5p, miR-181a-5p, miR-204-5p, miR-219-5p, miR-223-3p, miR-423-5p, miR-451a, miR-574-5p. They were selected out of 50-65 miRs reliably detectable in the human CSF [28,35,36,37,38,39,40] from the literature (PubMed, Dec. 31st 2017), considering their experimentally-validated or potential involvement in MS processes [15,16,28,36,39,41,42,43]. Next publications confirmed the reliability of our selection [5,6,17,44].

### 2.5. Detection of Inflammation-Related Protein in the CSF

Bio-Plex multiplex assays (Bio-Rad Laboratories, Hercules, CA, USA) were used for the quantification of the following cytokines, chemokines and growth factors: IL6, MIP1b, IL15, IL17, IL12_p70, FGFbasic, IL2, GM_CSF, IL9, IL4, IL7, G_CSF, VEGF, IL10, IL1β, IL13, eotaxin, TNFα, bbPDGF, RANTES, IL1ra, MIP1a, IL8, IP10, IL5, IFNγ, MCP1. Proteins below the detection sensitivity of the standard curve were considered as 0 pg/mL.

### 2.6. Statistical Analysis

Statistical analysis was performed using R software v3.6.3 (R Core Team 2020, https://www.R-project.org/) and Prism GraphPad 6.0. Data distribution was tested for normality with the Kolmogorov-Smirnov and Shapiro–Wilk tests. 

Hierarchical Clustering was used to divide miRNAs and biochemical parameters in groups of homogeneous entities [45]. In particular, for miRNA clustering, Pearson’s correlation coefficients of miRNA expression values were calculated and plotted in R using the corrplot package [44]. The correlation values were ordered according to the hierarchical clustering and the agglomeration method used was the “ward.D2”. MiRNA network analysis was performed and plotted with the igraph package [46] using Pearson’s coefficient values >0.5. For CSF biochemical parameters, clustering analysis was performed by means of hierarchical agglomerative clusters (complete linkage). The distance matrix for the hierarchical clustering was based on the Spearman correlation between variables. Next, we used the silhouette method to select the number of clusters (two clusters, see below) [47]. Finally, a principal analysis was performed separately for each of the two clusters and the first component (PC1) was used to evaluate the overall effect of each cluster on let7b-5p, similarly to [48]. Two patients with the score along PC1 of cluster 2 greater than three standard deviations above the mean and were removed from the analysis. The positive values on PC1 always corresponded to higher values in all parameters. Spearman correlation between let-7b-5p and single biochemical, demographic and clinical variables were also performed. Additionally, linear regression was computed to study the relationship between let7b-5p and the following predictor variables: the PC1 of each of the two clusters, age, gender and EDSS of MS cases and control subjects. Since the distribution of let7b-5p skewed, the log-transform of this variable was used for the regression analysis.

Differences between two groups were analyzed using Student’s t-test, Mann–Whitney test and Fisher’s exact test, as appropriate. Multiple comparisons were performed by Kruskal Wallis test followed by Dunn’s Multiple Comparison test as post hoc. *p*-values were corrected for multiple comparisons with the Benjamini and Hochberg method [49]. A FDR or a *p*-value < 0.05 was considered statistically significant.

## 3. Results

### 3.1. The Let-7 Family Regulates Crucial Processes Involved in MS Pathophysiology 

**Figure 1:**
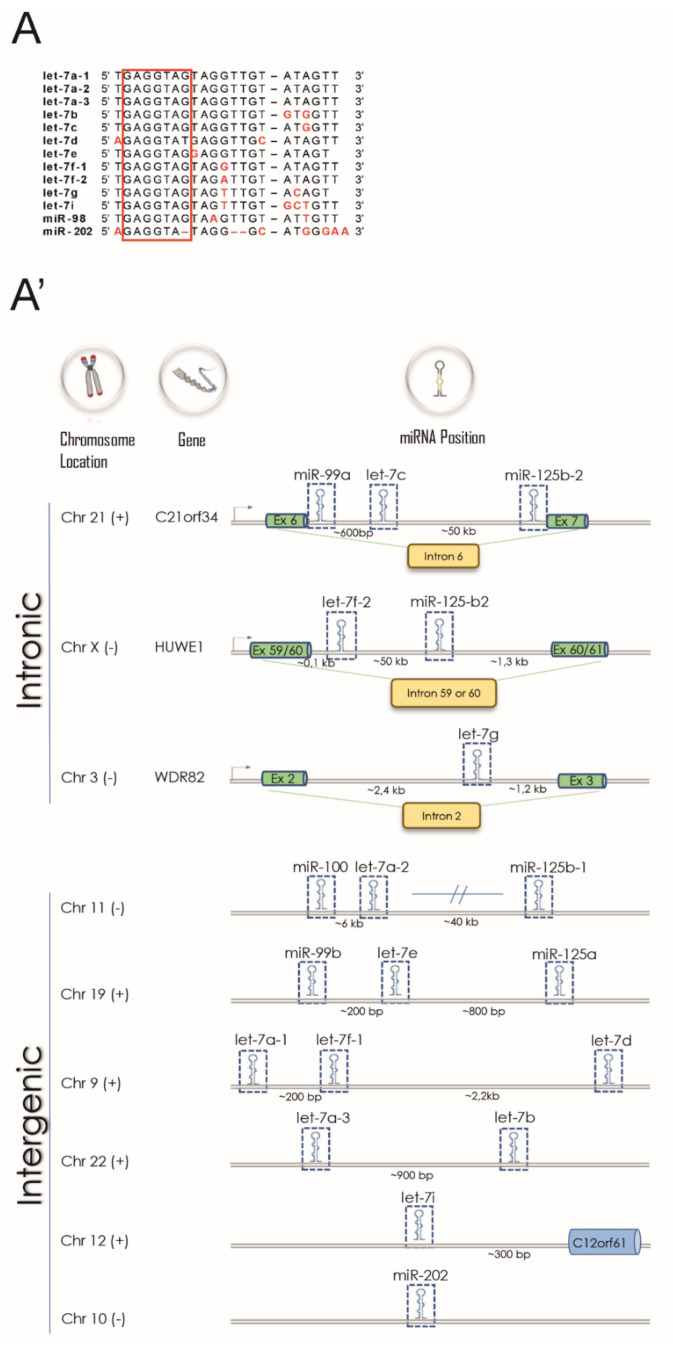

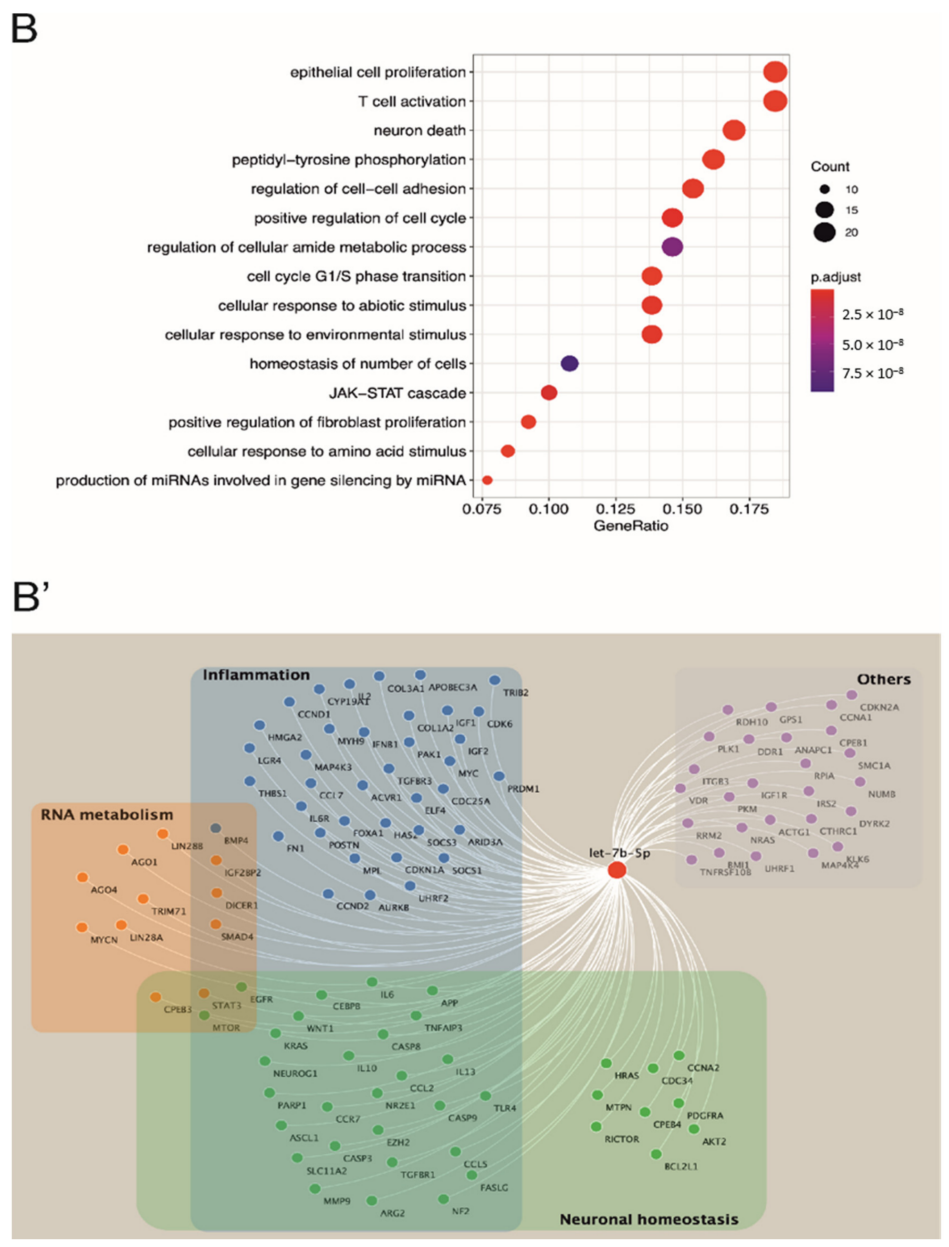
Let-7 family is a good candidate as a MS-associated miRNA. (**A**) DNA sequences alignment of the mature miRNAs part of the let-7 family. In black the nucleotides positions conserved among all members of the let-7 family. The seed sequence is indicated as a red box. (**A’**) Genomic organization of let-7 family genes in humans. Gene clusters are divided according to the genome organization (intronic or intergenic). Chromosome strands are indicated by (+) or (−). Figure information sourced from http://microrna.sanger.ac.uk/sequences/. (**B**) Functional analysis of experimentally validated target mRNAs of let-7 obtained from miRTarBase (http://mirtarbase.cuhk.edu.cn/php/index.php). The most represented Gene Ontology categories for target mRNAs are reported in the figure. Size dots are correlated with the number of genes that belong to a Gene Ontology category and dots are colored according to the Benjamini-Hochberg false discovery rate adjusted *p*-values from blue (higher *p*-adjusted) to red (lower *p*-adjusted). (**B’**) Network of let-7 targets that can be ascribed to three main processes involved in MS pathophysiology: inflammation (light blue rectangle); neuronal homeostasis (green rectangle); RNA metabolism (orange rectangle). Target mRNAs of let-7 involved in more than one process are represented into the rectangle overlapping zones. Targets participating in other pathways are grouped into a light violet rectangle (26 out of 130).

Both the sequences of miRNAs grouped in the let-7 family and their genomic organization are highly conserved among vertebrates [14]. Up to date, thirteen members of the family have been identified with specific chromosome locations as annotated in miRbase, the primary miRNA repository (http://www.mirbase.org/; [50]). Some of them are clustered together and are present with multiple copies in the human genome, like let-7f-1 and let-7f-2 (Figure 1A). Since all members of the family share the same seed sequence (nucleotides 2–8; Figure 1A’) they are able to regulate overlapping target mRNAs. 

Considering these aspects, we performed Gene Ontology (GO) analysis of experimentally validated target mRNAs of let-7 family (n = 132) recorded in miRTarBase [21] to evaluate its possible contribution to MS disease. We identified many significant GO categories by statistics for gene functional enrichment, including T cell activation (GO:0042110, 24/130, *p* adjusted = 1.27 × 10^−11^) and proliferation (GO:0045787, 19/130, *p* adjusted = 1.27 × 10^−9^) neuronal death (GO:0070997, 22/130, *p* adjusted = 3.99 × 10^−12^) cell response to environmental and nutrient stimuli as miRNA biosynthesis (GO:0035196, 10/130, *p* adjusted = 1.29 × 10^−10^) (Figure 1B). Interestingly, most of these different regulatory pathways have been demonstrated to participate in MS pathophysiology [3,51]. Moreover, we were able to assign the let-7 targets to three main processes that are crucial in MS pathophysiology (Figure 1B’), like inflammation (n = 74/132) [1,17], neuronal homeostasis (n = 50/132) [17,24,25] as well as RNA metabolism, including miRNA biosynthesis and function (n = 18/132) [5,26,27]. 

The encouraging results of our bioinformatic analysis based on the validated interactions between let-7 and target mRNAs prompted us to further investigate let-7 family in the context of MS.

### 3.2. Let-7b-5p Is a Possible Regulatory Hub of the Pattern of MS-Related miRNAs Circulating in the CSF

Considering that GO analysis revealed 18 experimental validated target mRNAs of let-7 family involved in miRNA metabolism, we evaluated the possible crosstalk between let-7 miRNAs and MS-linked miRNAs circulating in the CSF.

**Figure 2:**
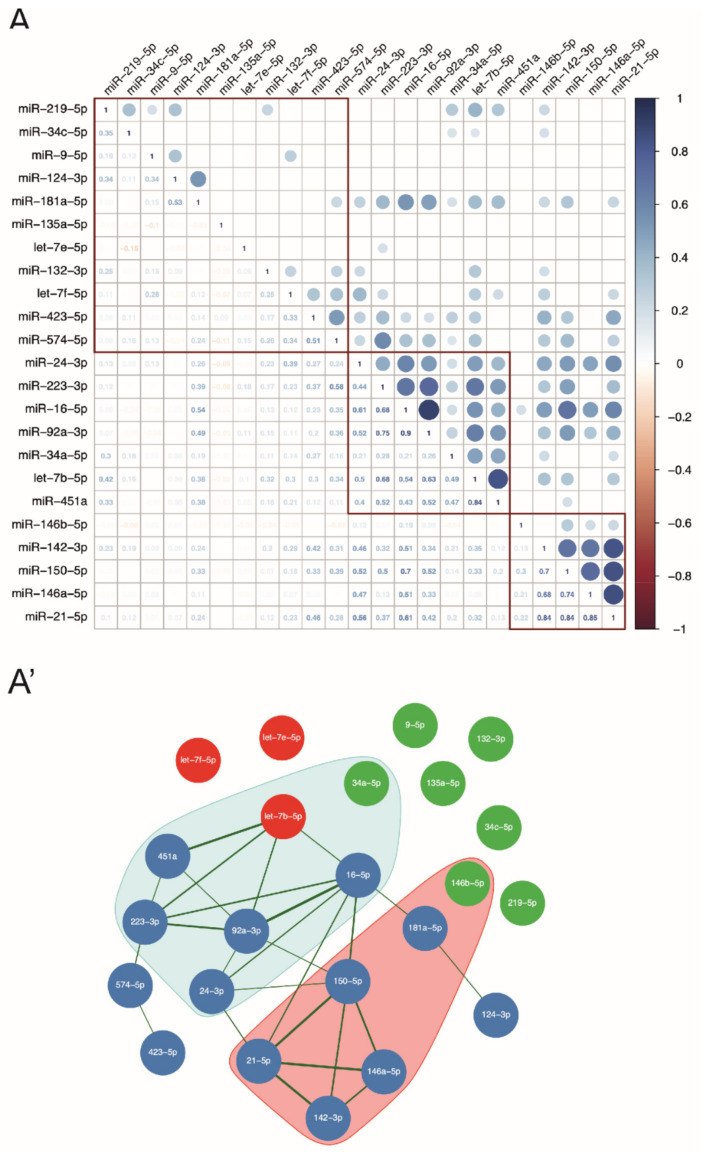
Let-7b-5p is a hub in the network of miRNAs in the CSF of patients with MS. (**A**) Heat map of Pearson’s correlation coefficients (r) between 23 miRNAs (relative to miR-204-5p, according to the ΔCt calculation) detected by qPCR in the CSF of the main cohort of patients (n = 166) at T0. In the upper triangle, r values of significant correlation (*p* < 0.05) were represented by coloured circles according to the scale (r > 0 is positive correlation, 0 no correlation and <−1 is negative correlation). In the lower triangle, r values are reported following the color code. Squares represent three different clusters identified by hierarchical clustering using the cutree R function with k = 3. Only statistically significant correlations with FDR < 0.05 are shown. (**A’**) Network representation of miRNA correlation. In red, there are detected members of the let-7 family (let-7b-5p, let-7e-5p and let-7f-5p). Blue nodes are other miRNAs relevant for MS, which correlate each other and/or with let-7b-5p (r ≥ 0.5). In green are miRNAs with r < 0.5. Pink and light blue areas represent, respectively, the first and the second correlation clusters, highlighted in panel A of the figure by the two lower squares.

To this aim, we enrolled 166 patients with MS (for details see Table 1), who underwent CSF withdrawal at diagnosis (T0) and we detected, by LNA-based qPCR experiments, the CSF levels of three representative members of the family, as let-7b-5p, let-7e-5p and let-7f-5p, together with other 21 miRNAs recently demonstrated to be CSF-enriched [5,36,38,40,41,52,53] as well as involved in MS [5,27,36,41]. First, GeNorm algorithm allowed us to identify miR-204-5p as the best internal reference in our qPCR raw data (Supplementary Figure S1), coherently with previous independent studies [35,36]. Then, by correlation analysis of miRNA levels relative to miR-204-5p, we observed that let-7b-5p levels in the CSF of MS patients highly correlated with an increased number of miRNAs compared to let-7e-5p or let-7f-5p, as shown by the correlation matrix (Figure 2A) and by the miRNA correlation network (Figure 2A’). More in detail, we observed strong and direct correlations (r > 0.5) between let-7b-5p and miR-451a (r = 0.84), miR-223-3p (r = 0.68), miR-92a-3p (r = 0.63) and miR-16-5p (r = 0.54). Interestingly, all these miRNAs have been implicated in MS as crucial regulators of the immune system [54,55,56,57,58,59] and/or CNS homeostasis [55,60,61,62,63]. Similar considerations can be made about miRNAs that were milder correlated with let-7b-5p (0.4 < r ≤ 0.5), like miR-24-3p and miR-34a-5p, or with the remyelination-related miR-219-3p [36], suggesting that multiple but common cellular sources participate to their release into the CSF [6,64]. Neither let-7e-5p or let-7f-5p showed any Pearson’s correlation with other miRNAs detected in the CSF of our cohort of patients (r ≥ ±0.4).

The results of miRNA-miRNA correlations combined with the functional analysis of miRNA-mRNA interaction revealed let-7b-5p as a possible fine-tuning regulator of miRNAs circulating in the CSF of patients with MS.

### 3.3. Let-7b-5p Is a Putative Anti-Inflammatory Regulator of the Complex Pathway of Soluble Factors Circulating in the MS CSF

Since most targets regulated by the let-7 family were involved in inflammatory response (Figure 1B,B’), we assessed the correlations between let-7b-5p levels in the CSF and possible protein players in MS inflammation, namely cytokines, chemokines and growth factors.

**Figure 3:**
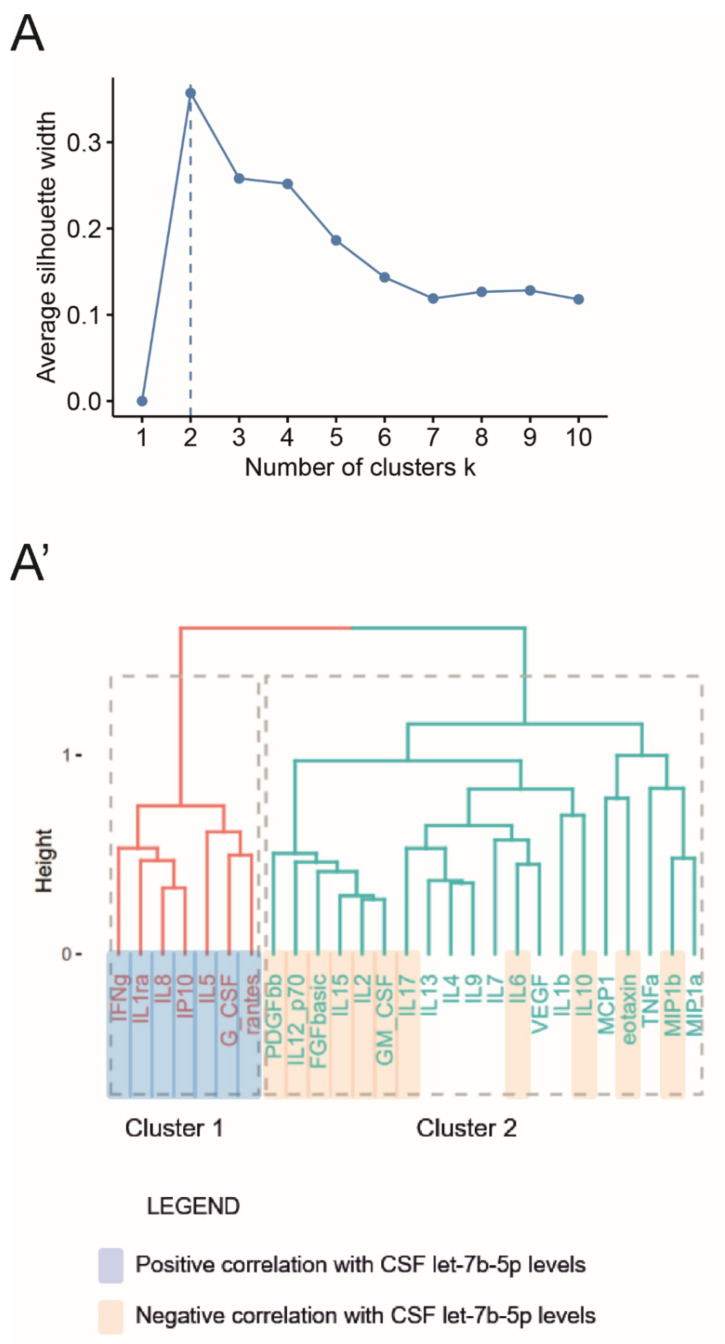
Let-7b-5p is an anti-inflammatory regulator of the complex pathway of soluble biochemical factors circulating in the MS CFS. By means of hierarchical cluster analysis, we divided in homogeneous groups, the 27 inflammation-related proteins (variables), quantified by multiple assays on 166 MS patients at T0. (**A**) We used the silhouette method to identify the optimal number of clusters, equal to two main clusters (**A’**) The result of this analysis was represented as a dendrogram: cluster 1 (red) with 7/27 inflammatory proteins (IFNγ, IL1ra, IL8, IP10, IL5, G_CSF, RANTES); cluster 2 (blue) including 20/27 inflammatory proteins (PDGFbb, IL12_p70, FGFbasic, IL15, IL2, GM_CSF, IL17, IL13, IL4, IL9, IL7, IL6, VEGF, IL1β, IL10, MCP1, eotaxin, TNFα, MIP1a, MIP1b).

Initially, we evaluated by multiplex assays the CSF levels of 27 inflammation-related factors in an extended cohort of patients with MS (n = 273, see Supplementary Table S1), including the main cohort of patients, whose we detected let-7b-5p level. Thus, we were able to perform a robust hierarchical clustering analysis using the silhouette method to select the appropriate number of clusters to consider (Figure 3A). The clustering analysis identified two different patterns of protein factors (Cluster 1: 7/27 inflammatory proteins; Cluster 2: 20/27 inflammatory proteins) as shown in the dendrogram (Figure 3A’). Similar results were obtained running the hierarchical cluster algorithm on the extended cohort values (data not shown).

We then computed the correlation between the CSF levels of each inflammatory protein and let-7b-5p. Importantly, as reported in Table 2 and Figure 3A’, we observed that let-7b-5p positively correlated with all members of the Cluster 1, and negatively correlated with most inflammation-related factors belonging to the Cluster 2, including also experimentally-validated targets or pathways of let-7 family, like IL6 [65,66,67,68], IL10 [15,69] and IL17 pathway [12].

### 3.4. The miR Let-7b-5p Is Reduced in the CSF of Patients with Progressive MS and Is Associated with Different Processes According to the Phase of the Disease

**Figure 4:**
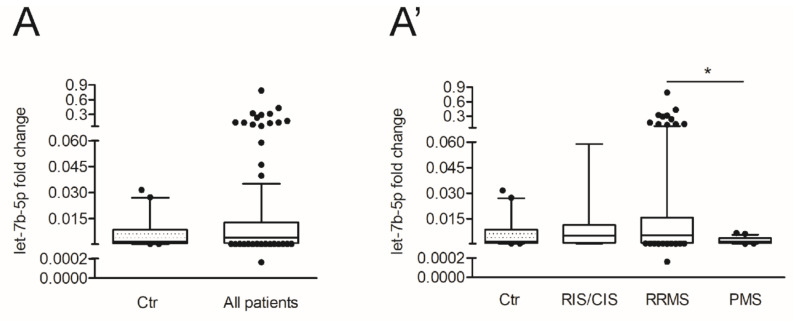
The levels of let-7b-5p are different according to diverse MS disease subtypes. (**A**) Box and whisker plots of let-7b-5p levels in the CSF, isolated from control subjects (Ctr) compared to all MS patients (Ctr, n = 20; All patients, n = 166; Mann-Whitney test, *p* > 0.05). (**A’**) Box and whisker plots of let-7b-5p levels in the CSF isolated from control subjects compared to patients separated in CIS/RIS, RRMS and PMS patients (Ctr, n = 20; CIS/RIS, n= 25; RRMS, n = 117; PMS, n = 24; Kruskal-Wallis test, * *p* < 0.05 RRMS vs. PMS). Data were normalized to miR-204-5p expressed as 2^−ΔCt let7b-5p-miR-204-5p^). Values are median of 2^−ΔCt^ with 10–90% percentiles (error bars) and 25–75% percentiles (open boxes).

To further investigate the involvement of let-7b-5p in MS pathology, we compared the miRNA levels in the CSF between control subjects and the main cohort of patients with MS. We observed a highly variable expression among the patients respect to control subjects (Ctr: n = 20; MS: n = 166; Mann–Whitney test, *p* > 0.05) (Figure 4A). Therefore, we asked whether the disease phase of the examined MS patients could highlight more remarkable differences in terms of expression levels of let-7b-5p. To this aim, we stratified patients into three groups based on the disease subtypes, CIS/RIS: (n = 25), RRMS (n = 117) and PMS (n = 24) (see Table 1), and we observed that the level of let-7b-5p was significantly reduced in PMS patients in comparison to RRMS (Kruskal-Wallis test, *p* < 0.05) (Figure 4A’). Since the variability and the median values of CSF let-7b-5p levels were similar between the patients with CIS/RIS and RRMS, they were grouped together in the following statistical correlations with different aspects of the disease, and they were referred to as patients with non-progressive MS (non-PMS).

Central and peripheral inflammation was the first aspect of the disease that we examined in association with CSF let-7b-5p in stratified patients, considering our previous results (Table 2 and Figure 3) obtained on all patients of the main cohort as well as the recent evidence about the crucial contribution of cytokines and growth factors released from infiltrating autoreactive T cells to neuronal damage in MS [70,71]. Similar to what we performed on all patients of the main cohort, we correlated CSF let-7b-5p levels with the CSF amount of inflammation-related protein factors in the subgroups of patients. In particular, we noticed that all the correlations observed in the main cohort were maintained in the non-PMS subgroup (CIS/RIS/RRMS; Table 3). 

**Figure 5:**
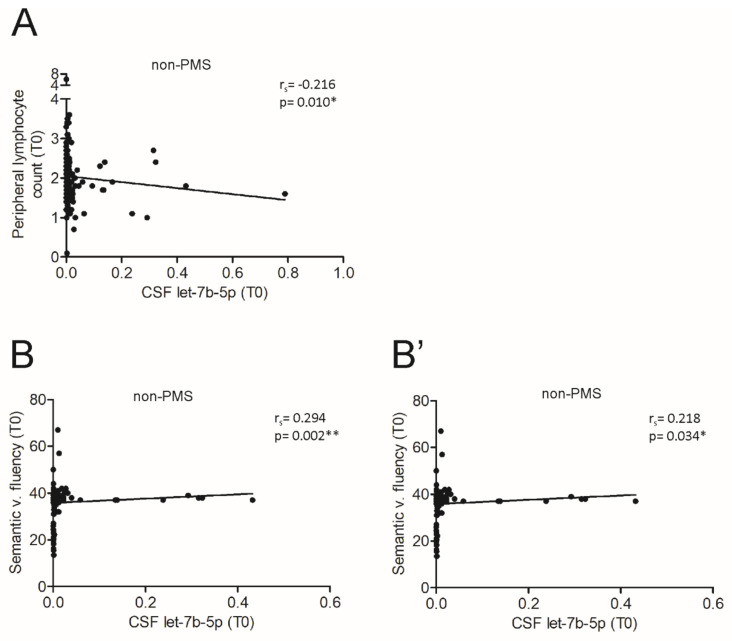
The correlations with inflammation and cognitive performances revealed a putative protective role of let-7b-5p in non-progressive phase. (**A**) Correlation plot between let-7b-5p levels and the count of peripheral T cells of non-PMS (n = 140) at T0. A negative correlation was observed (Spearman’s correlation: r_s_ = −0.216, * *p* < 0.01). (**B**,**B’**) Correlation plot between let-7b-5p levels and Scheme 106. and Phonemic (n = 95) verbal fluency of non-PMS patients at T0. A positive correlation was observed in both executive and categorical memory functions (Spearman’s correlation, B: r_s_ = −0.294, *p* < 0.01; B’: r_s_ = 0.218, ** *p* < 0.05).

Then, we evaluated the peripheral inflammation by counting the number of lymphocytes in the blood at T0. Interestingly, we found that the count of peripheral lymphocytes negatively correlated with let-7b-5p levels in the CSF of patients with non-PMS (Spearman’s correlation: r_s_ = −0.216, *p* < 0.05; Figure 5A), in accordance with the inverse correlation with central inflammation.

To explore the clinical implication of this consideration, we assessed the possible link between the demographic or clinical parameters at both T0 and Tf1 (age, sex, disease duration, EDSS and PI) and let-7b-5p levels in non-PMS CSF. No significant associations were observed (data not shown). On the contrary, in a subset of patients, let-7b-5p levels was directly correlated with the cognitive performances related to executive functions (Semantic verbal fluency; n = 106, Spearman’s correlation: r_s_ = 0.294, *p* < 0.01, Figure 5B) and categorial memory functions (Phonemic verbal fluency; n = 95, Spearman’s correlation: r = 0.218, *p* < 0.05, Figure 5B’), suggesting a protective role for the miRNA in the neuronal compartment linked to an anti-inflammatory action.

**Figure 6:**
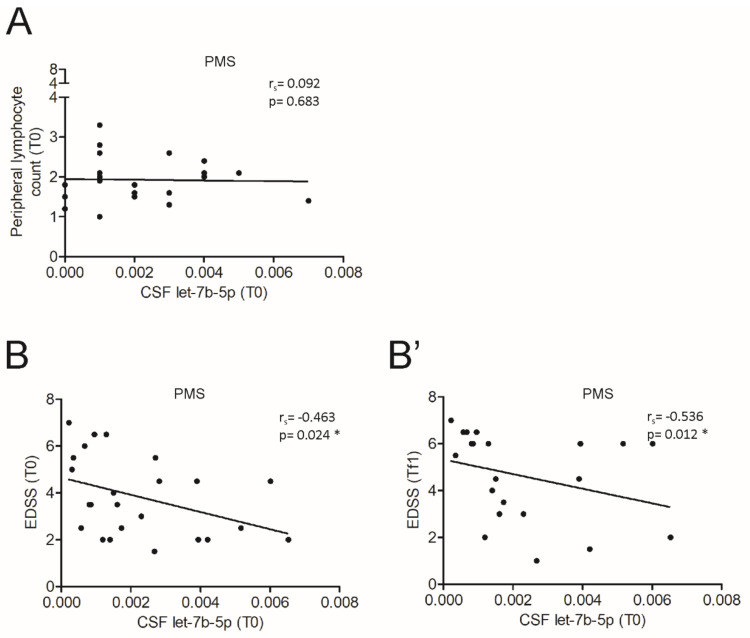
The CSF level of let-7b-5p correlates with disease severity in MS progressive phase. (**A**) Correlation plot between let-7b-5p levels and the count of peripheral T cells of PMS patients (n = 22, **A**) at T0. (**B**,**B’**) Correlation plot between let-7b-5p levels and EDSS of PMS patients at T0 (n = 24, **B**) and after a follow-up period ((Tf1), n = 21, **B’**). A negative correlation was observed at both T0 (Spearman’s correlation, Spearman’s r = −0.463, * *p* < 0.05) and Tf1 (Spearman’s correlation, Spearman’s r = −0.536, * *p* < 0.05).

Not surprisingly, no correlations were found between inflammatory parameters and let-7b-5p levels in patients with PMS (Peripheral lymphocyte count, Spearman’s correlation: r_s_ = 0.092, n. s.; Figure 6A), with exception of IL5, RANTES and G_CSF (Table 4). According to the neurodegenerative phase of disease, we found that let-7b-5p negatively correlated with the clinical disability in terms of EDSS at both onset (T0; Spearman’s correlation: r_s_ = −0.463, *p* < 0.05) and after a follow-up period (Tf1; Spearman’s correlation: r_s_ = −0.536, *p* < 0.05) in PMS patients (Figure 6B,B’), while no significant correlations were observed for the other clinical parameters (data not shown).

Moreover, no changes in the CSF level of let-7e-5p and let-7f-5p were found in the main cohort, considering both all patients and stratifying them for MS subtype (Supplementary Figure S2). Similarly, let-7e-5p (Supplementary Figure S3) and let-7f-5p (Supplementary Figure S4) were not correlated with any inflammatory or clinical parameters in each phase of the disease, highlighting the specificity of the results obtained on let-7b-5p.

Finally, to study the relationship between let-7b-5p and multiple parameters relevant to MS course, we performed linear regression analysis in both non-PMS and PMS conditions. As predictor variables, we considered age, genders, EDSS and the CNS inflammatory milieu evaluated by performing the principal component analysis and saving the first component (PC1) of two clusters of the inflammatory mediators described before (Figure 3). In the non-PMS group, we found a positive association between let-7b-5p and cluster 1, and negative association with cluster 2. Both were statistically significant (Table 5). We replicated the same analysis for the PMS group (Table 5) and control subjects (data not shown). Anyway, we did not find a significant association between either cluster of inflammatory factors and let-7b-5p in neither group. Conversely, both age (estimate = 0.048, *p* < 0.05) and EDSS (estimate = −0.386, *p* < 0.01) were significantly associated with let-7b-5p in the PMS group (Table 5), similar to what observed by single correlation analyses.

Overall, these data suggest that let-7b-5p, likely derived from diverse cellular sources, can participate in the different processes running in the CNS of patients with MS depending on the phase of the disease.

## 4. Discussion

In the last few years, circulating miRNAs have been proposed as potential diagnostic and prognostic biomarkers or even as therapeutic targets for various diseases, including CNS disorders like MS [6,17,72,73]. The let-7 family regulates many target mRNAs by participating in crucial processes for MS pathophysiology (neuronal homeostasis [17,24,25], inflammation [1,17] and miRNA metabolism [5,26,27], as in Figure 1B,B’). Notwithstanding, the impact of let-7 family on MS disease has been scarcely investigated, especially in humans.

In this context, we explored three representative members of the let-7 family (let-7b-5p, let-7e-5p, let-7f-5p) in terms of CSF abundance and correlation with other 21 MS-related miRNAs as well as potential implications in MS disease. Although all let-7 miRNAs have the possibility to control miRNA biogenesis and functioning because they share the same repertoire of target mRNAs with a role in miRNA metabolism, we specifically identified let-7b-5p as a possible hub of a network of seven miRNAs highly linked to MS [54,55,56,57,58,59,60,61,62,63,74]. Neither let-7e-5p or let-7f-5p showed such strong correlations with other detected miRNAs in the CSF, suggesting that the timing and cellular sources are as important as the target mRNA subset in MS regulation. In particular, let-7b-5p directly correlated with protective miRNAs, like miR-451a, miR-219-3p and miR-223-3p. MiR-451a is known to inhibit the nuclear factor-kappa B (NF-κB)-mediated proinflammatory response [74] and the microglia activation by repressing, together with let-7b-5p [75], the toll like receptor 4 (TLR4) [60]. MiR-219-3p is necessary for myelination and its absence in the CSF correlates with MS diagnosis [36]. MiR-223-3p can exert a neuroprotective action [55,61], although preclinical studies demonstrated that miR-223 knockout mice develop a less severe experimental MS [56]. Also, 92a-3p, which has an anti-excitotoxic role in neurons [63] but pro-inflammatory effects in the immune system [57], was in the network with let-7b-5p together with miR-34a-5p, showing an opposite role according to the cellular context of expression [76,77]. The last two miRNAs in cluster with let-7b-5p were miR-16-5p and miR-24-3p, both upregulated in the peripheral or/and central compartments of patients with MS [58,78] and associated with disability accumulation [58,59,78].

Both let-7 target analysis and miRNA correlation network in MS CSF highlighted let-7b-5p as a “meta-miRNA” able to regulate different MS-linked miRNAs in different cellular contexts, coherently with previous studies. Indeed, let-7b-5p has been observed in peripheral blood [18] or derivatives [19,79] as well as in the CNS cells [16,80], confirming its multiple functions.

Furthermore, we evaluated an additional regulatory aspect of let-7b-5p by analyzing its possible interaction with twenty-seven MS-related protein factors circulating in the CSF of patients with MS. The inflammatory milieu associated with MS showed a double pattern of opposite correlations with the CSF levels of let-7b-5p. We speculated that the soluble mediators positively correlating with let-7b-5p (cluster 1, Figure 3A’) could be involved in the miRNA induction and/or could act synergically in the same pathways. On the contrary, several direct and indirect experimentally-validated target mRNAs of let-7 family are negatively correlated with the let-7b-5p levels (cluster 2, IL6 [65,66,67,68]; IL10 [15,69]; IL17 pathway [12]). Considering the complex system of multiple feedback loops regulating the CNS homeostasis, these correlations suggested that the let-7b-5p might be considered as pleiotropic modulator of CSF molecules with possible protective implication in MS course, although let-7b-5p cannot be univocally ascribed as an absolute anti-inflammatory factor.

The putative protective role of let-7b-5p was further supported by our observation reporting lower levels of circulating let-7b-5p in CSF of patients with PMS compared to RRMS patients. In the relapsing-remitting phase of the disease, we hypothesize that let-7b-5p might be triggered by inflammatory insults, through the IFNγ and IP10 pathway activation as well as IL8, G_CSF and RANTES signals, in the attempt to counteract the pro-inflammatory action of the soluble mediators such as IL2, IL6, IL12 (p70), IL17, GM_CSF, MIP1b [81,82]. Coherently with this speculation, the CSF levels of let-7b-5p were inversely correlated with peripheral inflammation, measured by blood lymphocytes count and directly correlated with a better cognitive performance.

In the progressive phase of MS, the inflammation is less evident and neurodegenerative events are more prominent [3]. Indeed, the CSF let-7b-5p levels were reduced and few direct correlations with some of the inflammation-related proteins, such as IL5, RANTES and G_CSF were showed, possibly promoting a residual expression of the miRNA. Furthermore, let-7b-5p levels were negatively correlated with the severity of the disease, assessed by EDSS evaluation at both the CSF withdrawal and after 1-year follow-up, revealing a potential neuroprotective action of the miRNA in this context.

Our observations were also confirmed by using a multivariable approach, which underlined a possible anti-inflammatory and neuroprotective action of let-7b-5p specifically in MS condition, since no association in the control subjects’ group were found.

Let-7e-5p and let-7f-5p were not associated with any of the considered aspects both in non-PMS or in PMS condition. It cannot be excluded a contribute of other members of let-7 family, as let-7-g and let-7i, recently found to be involved in MS [12,13] although their expression seems to be limited to peripheral cells and their levels in the CSF are generally lower than let-7b-5p [37,38]. However, further experiments are needed to elucidate this aspect.

All together our investigations suggest let-7b-5p as a protective factor for MS course, in terms of both inflammation and clinical disability. The combination of our proposed bioinformatics strategy with miRNA-mRNA regulatory network building and integrated biochemical approach may help to better understand the mechanism underlying MS. Considering that let-7b-5p levels have been recently associated with good response to IFNβ treatment [19], it is reasonable to consider let-7b-5p as a potential biomarker. The next stage is to further validate our findings using larger cohorts of patients and datasets as well as a longer follow-up period in order to deepen the mechanism at the basis of the let-7b-5p in MS inflammation and neurodegeneration.

## Figures and Tables

**Table 1:** Demographic and clinical features of the main cohort of patients with MS and control subjects.

Patients’ Group					
Variable	Control Subjects	All Patients	CIS/RIS	RRMS	PMS
N	20	166	25	117	24
Age	46.1 (29.7–53.3)	39.8 (29.7–47.9)	41.0 (32.9–42.9)	35.6 (27.7–46.7)	48.6 (39.6–55.5)
Gender: F	7 (35.0%)	57 (34.3%)	6 (24%)	38 (32.5%)	13 (54.2%)
Oligoclonal Bandingy/n/NA	/	27 (17.2%)	10/14/1 (41.7%)	15/95/7 (13.6%)	2/21/1 (8.7%)
Disease activityy/n/NA	/	81 (48.8%)	10/14/1 (41.7%)	67/43/7 (60.9%)	4 (16.7%)
EDSS	/	2.0 (1.0–3.0)	1.0 (1.0–2.0)	1.5 (1.0–3.0)	3.5 (2.2–5.2)
Disease Duration	/	12.4 (2.4–37.7)	2.8 (1.6–4.7)	12.9 (1.8–38.9)	24.3 (12.3–61.3)
PI (T0)	/	0.2 (0.0–0.6)	0.6 (0.2–1.0)	0.1 (0.0–0.6)	0.2 (0.0–0.2)
let-7b-5p	0.002 (0.001–0.009)	0.004 (0.001–0.012)	0.005 (0.001–0.010)	0.005 (0.001–0.015)	0.002 (0.001–0.003)
let-7e-5p	0.003 (0.001–0.010)	0.002 (0.001–0.006)	0.002 (0.000–0.01)	0.002 (0.001–0.006)	0.004 (0.002–0.006)
let-7f-5p	0.004 (0.003–0.007)	0.004 (0.002–0.006)	0.004 (0.003–0.007)	0.004 (0.002–0.006)	0.004 (0.002–0.005)

Data are median and 25th-75th percentiles. Abbreviations: MS = Multiple Sclerosis; CIS/RIS = Clinically Isolated Syndrome/Radiologically Isolated Syndrome; RRMS = Relapsing-Remitting MS; PMS = Progressive MS; CSF = Cerebrospinal Fluid; EDSS = Expanded Disability Status Scale; F = Female; y/n/NA = yes/no/Not Available; PI(T0) = Progression Index at T0.

**Table 2:** Correlation between the levels of let-7b-5p and biochemical factors circulating in the CSF of patients with MS at T0.

		corr	*p* Value	*p* Adjusted	
Cytokines	IFNγ	0.224	0.004	0.006	*Cluster 1*
	IL1ra	0.311	<0.001	<0.001	
	IL5	0.308	<0.001	0.0002	
	IL2	−0.386	<0.001	<0.001	*Cluster 2*
	IL6	−0.277	<0.001	0.001	
	IL10	−0.257	0.001	0.002	
	IL12_p70	−0.294	<0.001	<0.001	
	IL15	−0.348	<0.001	<0.001	
	IL17	−0.247	0.001	0.002	
	GM_CSF	−0.420	<0.001	<0.001	
Chemokines	IL8	0.311	<0.001	<0.001	*Cluster 1*
	IP10	0.379	<0.001	<0.001	
	Rantes	0.392	<0.001	<0.001	
	Eotaxin	−0.234	0.002	0.004	*Cluster 2*
	MIP1b	−0.237	0.002	0.004	
Growth Factors	G_CSF	0.387	<0.001	<0.001	*Cluster 1*
	bFGF	−0.340	<0.001	<0.001	*Cluster 2*
	PDGF bb	−0.307	<0.001	<0.001	

Abbreviatons: IFN = Interferon; IL = Interleukin; GM_CSF = Granulocyte Macrophage Colony-Stimulating Factor; IP-10 = *Interferon γ* inducible *Protein 10*; MIP1b = Macrophage Inflammatory Protein 1; G_CSF = *Granulocyte* Colony-StimulatiFactor; bFGF = Basic Fibroblast Growth Factor; PDGFbb = *Platelet-Derived Growth Factor-*bb.

**Table 3:** Correlation between the levels of let-7b-5p and biochemical factors circulating in the CSF of patients with non-PMS at T0.

		corr	*p* Value	*p* Adjusted	
Cytokines	IFNγ	0.249	0.003	0.005	*Cluster 1*
	IL1ra	0.317	<0.001	<0.001	
	IL5	0.287	0.001	0.001	
	IL2	−0.383	<0.001	<0.001	*Cluster 2*
	IL6	−0.293	<0.001	0.001	
	IL10	−0.266	0.001	0.003	
	IL12_p70	−0.283	0.001	0.001	
	IL15	−0.338	<0.001	<0.001	
	IL17	−0.248	0.003	0.005	
	GM_CSF	−0.416	<0.001	<0.001	
Chemokines	IL8	0.309	<0.001	0.001	*Cluster 1*
	IP10	0.388	<0.001	<0.001	
	Rantes	0.364	<0.001	<0.001	
	Eotaxin	−0.231	0.006	0.009	*Cluster 2*
	MIP1b	−0.226	0.007	0.010	
Growth Factors	G_CSF	0.381	<0.001	<0.001	*Cluster 1*
	bFGF	−0.324	<0.001	<0.001	*Cluster 2*
	PDGF bb	−0.299	<0.001	0.001	

Abbreviatons: IFN = Interferon; IL = Interleukin; GM_CSF = Granulocyte Macrophage Colony-Stimulating Factor; IP-10 = *Interferon γ* inducible *Protein 10*; MIP1b = Macrophage Inflammatory Protein 1; G_CSF = *Granulocyte* Colony-Stimulating Factor; bFGF = Basic Fibroblast Growth Factor; PDGFbb = *Platelet-Derived Growth Factor-*bb.

**Table 4:** Correlation between the levels of let-7b-5p and biochemical factors circulating in the CSF of patients with PMS at T0.

		corr	*p* Value	*p* Adjusted	
Cytokines	IL5	0.567	0.004	0.035	*Cluster 1*
Chemokines	Rantes	0.628	0.001	0.028	
Growth Factors	G_CSF	0.587	0.003	0.035	

Abbreviations: IL, Interleukin; G_CSF: *Granulocyte* Colony-Stimulating Factor.

**Table 5:** Results of the multiple regression analysis for let-7b-5p with EDSS, age and gender in non-PMS and PMS patients.

		Estimate	Std. Error	T-Value	Pr(>|t|)
non-PMS	PC1 cluster 1	0.291 −0.233 −0.111 −0.012 −0.185	0.010 0.070 0.120 0.012 0.316	2.985 −3.306 −0.930 −0.976 −0.587	0.003 0.001 0.354 0.331 0.558
	PC1 cluster 2				
	EDSS				
	Age				
	Gender				
PMS	PC1cluster 1	0.177	0.087	2.045	0.057
	PC1cluster 2	−0.042	0.099	−0.427	0.674
	EDSS	−0.386	0.109	−3.542	0.002
	Age	0.048	0.019	2.526	0.021
	Gender	−0.143	0.347	−0.412	0.685

PC1: Principal Component 1; EDSS = Expanded Disability Status Scale; non-PMS = CIS/RIS/RR; PMS = Progressive MS.

## Data Availability

The data presented in this study are available on request from the corresponding author.

## Supplementary Materials

The following are available online at https://www.mdpi.com/2073-4409/10/2/330/s1, Figure S1: Quantification cycle (Cq) of miRNAs detected in the CSF of patients with MS. Figure S2: The let-7e-5p and let-7f-5p levels in MS disease subtypes. Figure S3: Correlations between let-7e-5p levels, peripheral inflammation and EDSS of patients with non-progressive and progressive MS. Figure S4: Correlations between let-7f-5p levels, peripheral inflammation and EDSS of patients with non-progressive and progressive MS. Table S1: Demographic and clinical characteristics of patients with MS included in the extended cohort.
