# Supplementary material for: The microRNA let-7b-5p Is Negatively Associated with Inflammation and Disease Severity in Multiple Sclerosis
Source: Cells. 2021 Feb 5;10(2):330. doi: 10.3390/cells10020330 (PMC7915741; doi:10.3390/cells10020330)
Supplement: Supplementary file 1 [file cells-10-00330-s001.zip › Supplementary Figures and Tables/Supplementary Tables.docx]

| **Supplementary Table 1: Demographic and clinical characteristics of patients with MS included in the extended cohort** | | | | | | | | |  |
| --- | --- | --- | --- | --- | --- | --- | --- | --- | --- |
|  | |  | |  | | ***Patients’ Group*** | |  |  |
| ***Variable*** | ***Total*** | | ***CIS/RIS*** | | ***RRMS*** | | ***PMS*** | |  |
| N | 273 | | 49 | | 180 | | 44 | |  |
| Age | 40.4 (29.4-49.9) | | 41 (29.4-49.5) | | 36.7 (27.3-47.0) | | 50.6 (44.0-59.4) | |  |
| Gender: F | 95 (34.8%) | | 13 (26.5%) | | 60 (33.3%) | | 22 (50.0%) | |  |
| Oligoclonal Banding  y/n/NA | 53/209/11 (20.2%) | | 21/26/2 (44.7%) | | 25/147/8 (14.5%) | | 7/36/1 (16.3%) | |  |
| EDSS | 2.0 (1.0-3.0) | | 1.0 (0.0-2.0) | | 1.5 (1.0-2.5) | | 3.5 (2.5-4.9) | |  |
| Disease Duration | 12.4 (2.8-43.6) | | 3.4 (2.0-8.5) | | 12.3 (2.3-37.7) | | 36.5 (12.5-111.9) | |  |
| PI (T0) | 0.2 (0.0-0.5) | | 0.5 (0.1-1.0) | | 0.1 (0.0-0.6) | | 0.1 (0.0-0.2) | |  |
| Data are median and 25^th^-75^th^ percentiles. Abbreviations: MS = Multiple Sclerosis;  CIS/RIS = Clinically Isolated Syndrome/Radiologically Isolated Syndrome; RRMS = Relapsing-Remitting MS;  PMS = Progressive MS; EDSS = Expanded Disability Status Scale; F = Female;  y/n/NA = yes/no/Not Available; PI(T0) = Progression Index at T0. | | | | | | | | |  |
