# Supplementary figures and images for: The microRNA let-7b-5p Is Negatively Associated with Inflammation and Disease Severity in Multiple Sclerosis
Source: Cells. 2021 Feb 5;10(2):330. doi: 10.3390/cells10020330 (PMC7915741; doi:10.3390/cells10020330)

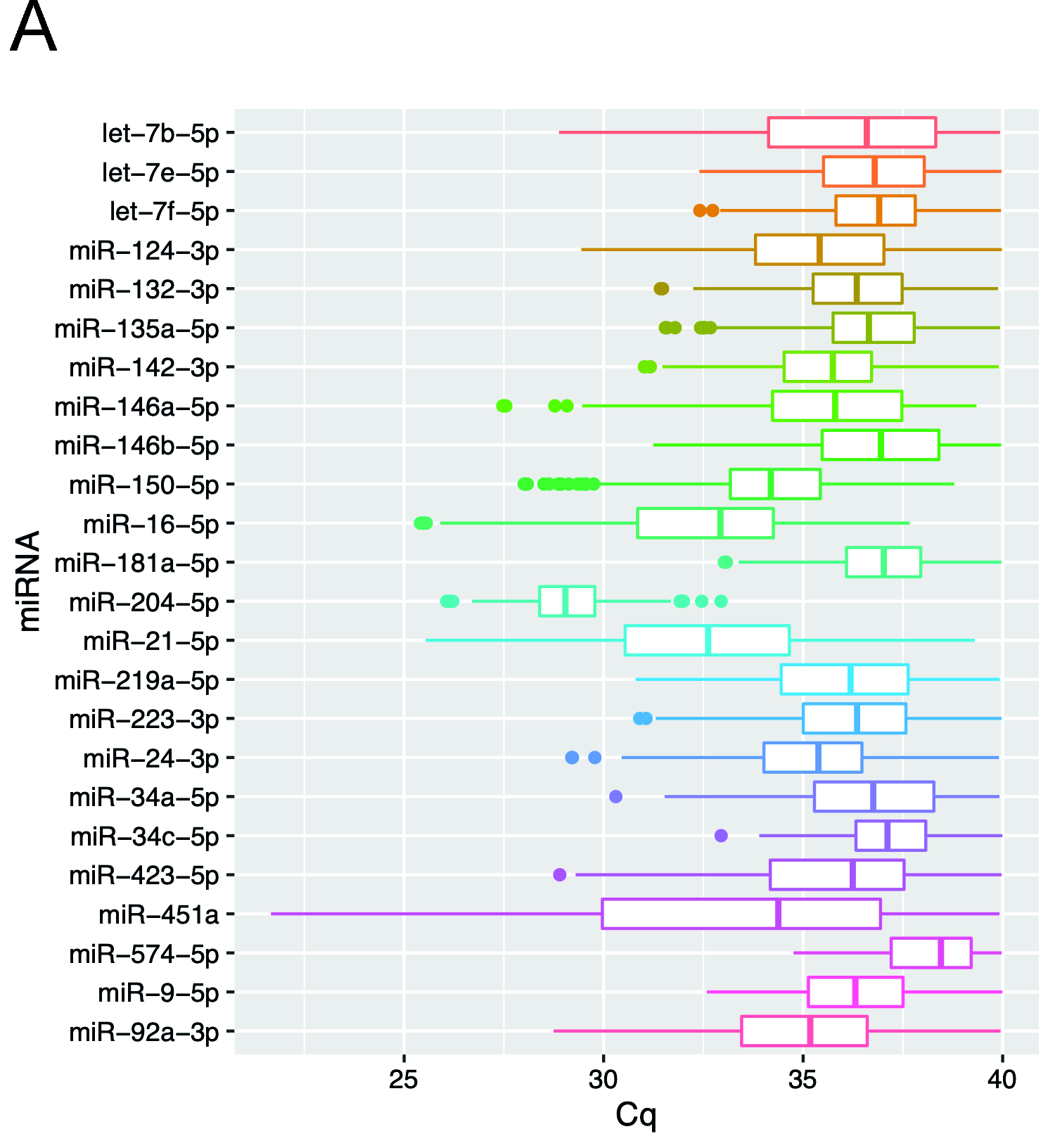

Supplement: Supplementary file 1 [file cells-10-00330-s001.zip › Supplementary Figures and Tables/Supplementary Figure 1.tif]

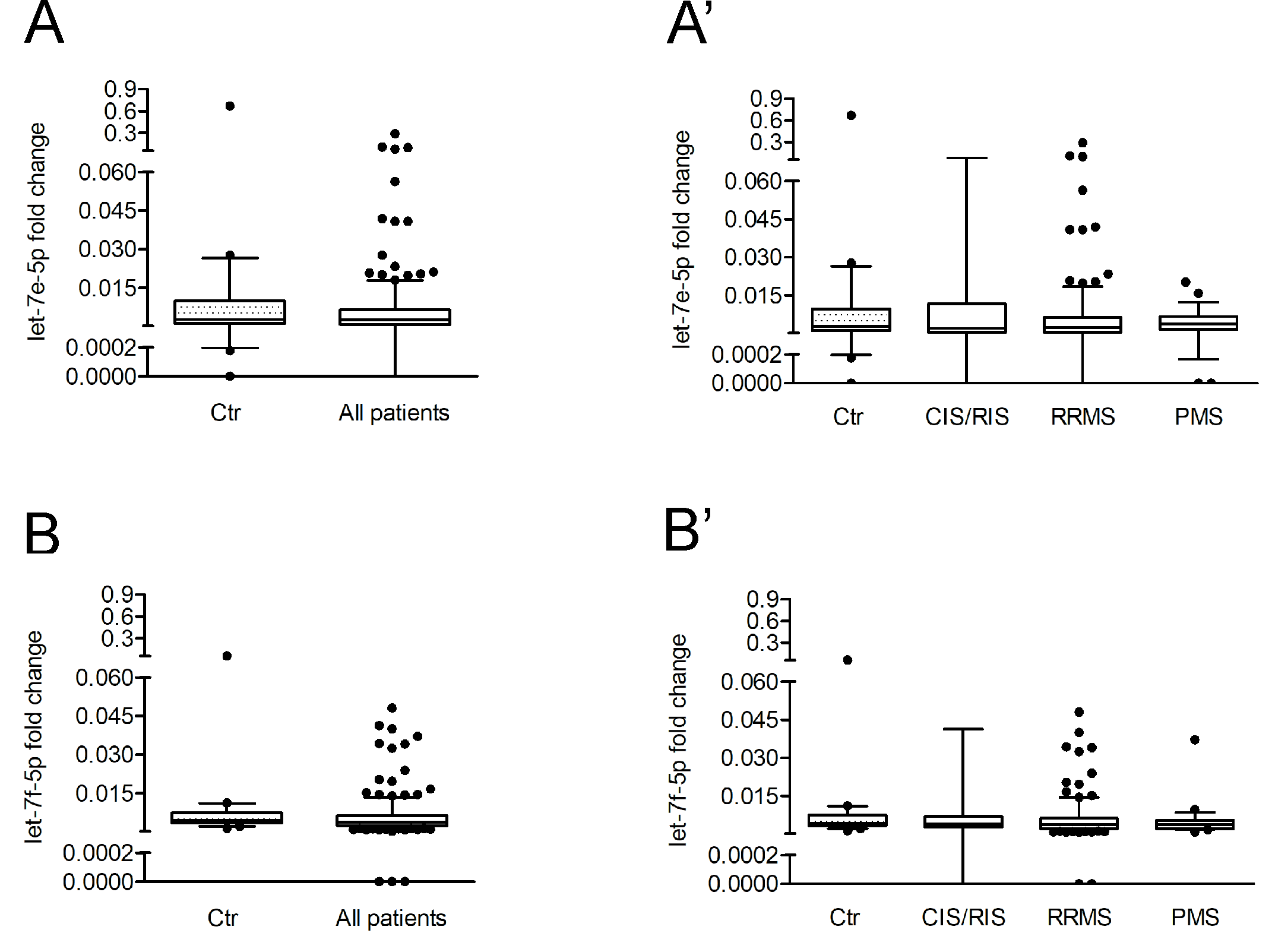

Supplement: Supplementary file 1 [file cells-10-00330-s001.zip › Supplementary Figures and Tables/Supplementary Figure 2.tif]

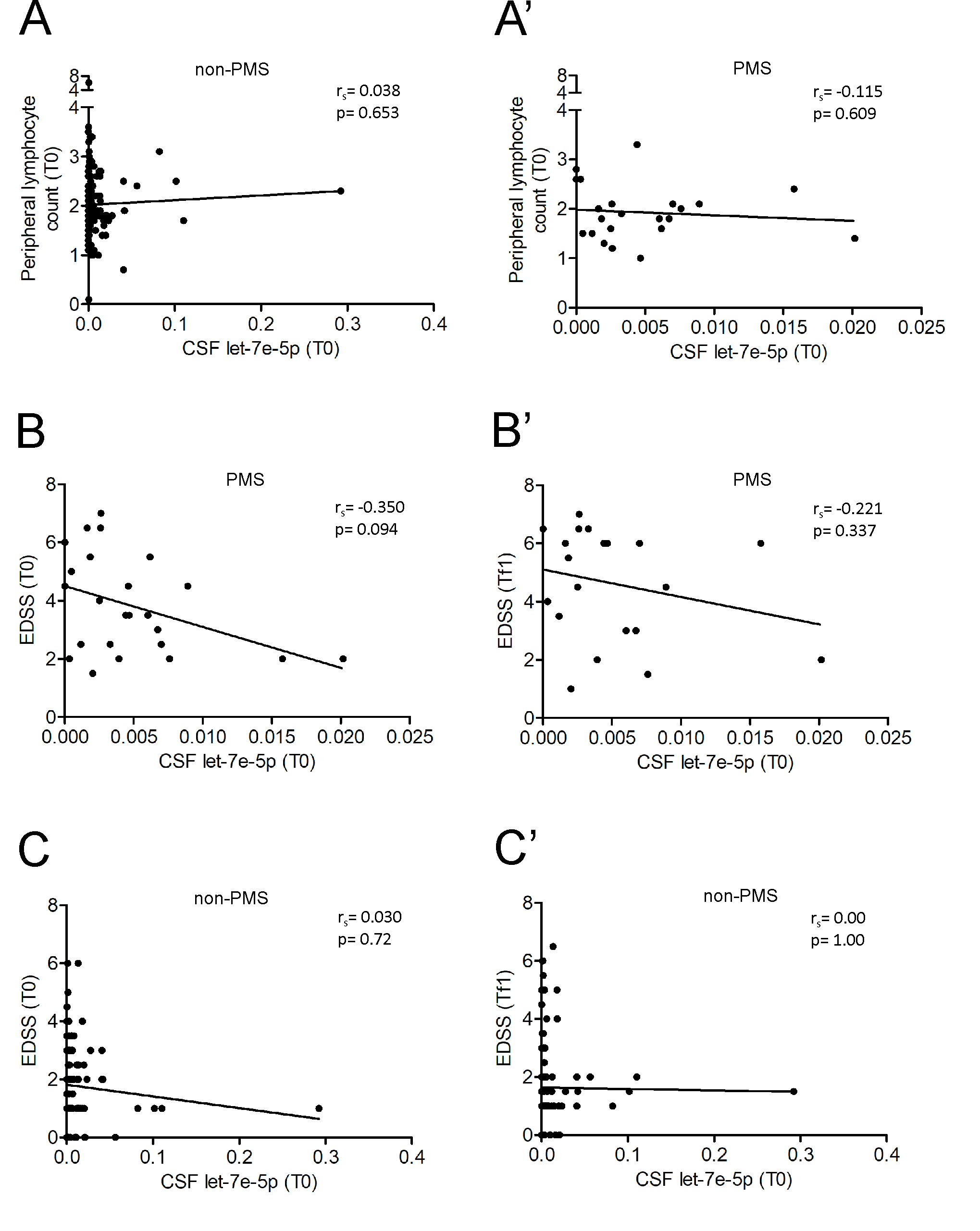

Supplement: Supplementary file 1 [file cells-10-00330-s001.zip › Supplementary Figures and Tables/Supplementary Figure 3.tif]

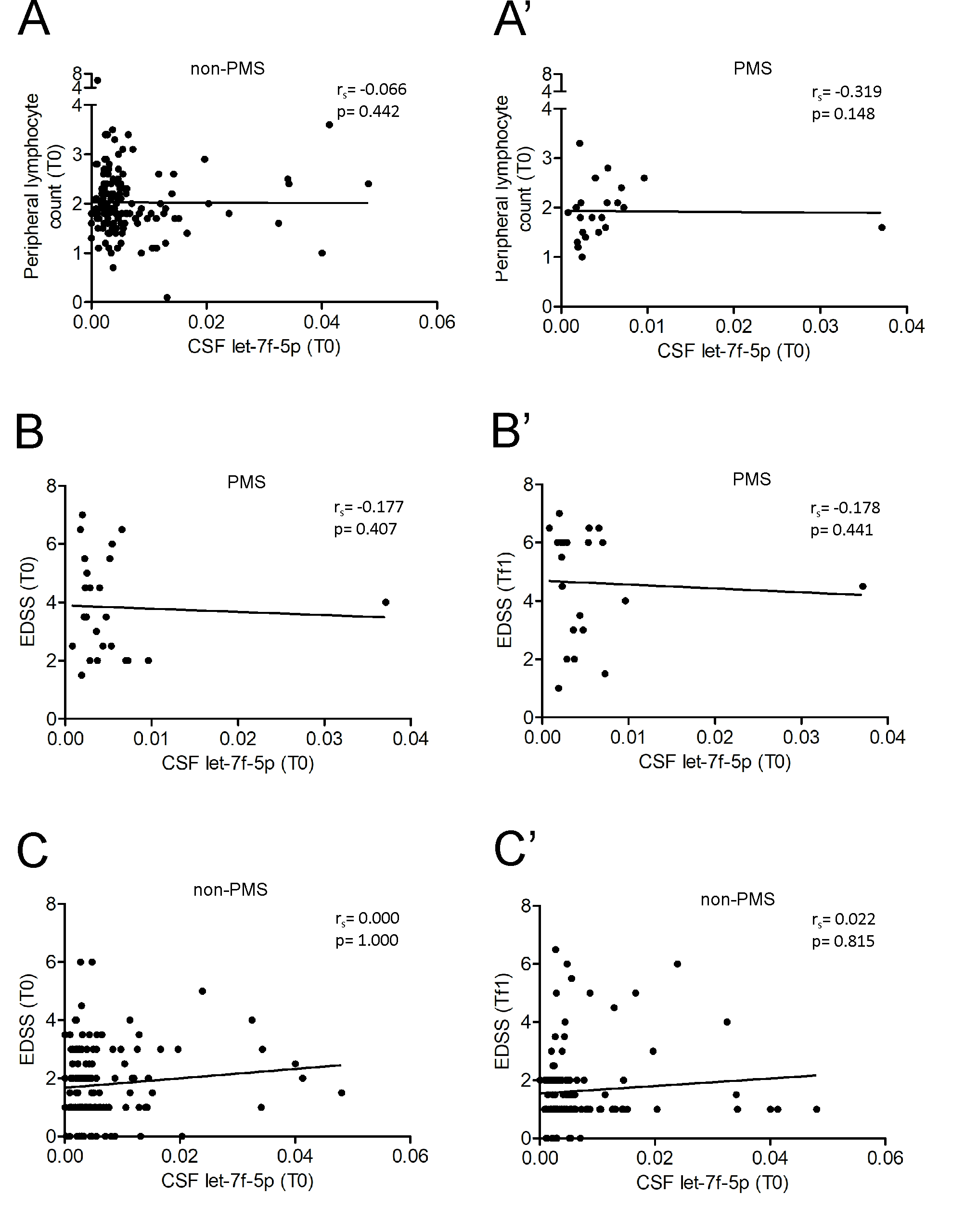

Supplement: Supplementary file 1 [file cells-10-00330-s001.zip › Supplementary Figures and Tables/Supplementary Figure 4.tif]
